# Individual and Group-Based Engagement in an Online Physical Activity Monitoring Program in Georgia

**DOI:** 10.5888/pcd15.170223

**Published:** 2018-06-07

**Authors:** Matthew Lee Smith, Nicholas K. Durrett, Maria Bowie, Alison Berg, Bryan A. McCullick, Alexander C. LoPilato, Deborah Murray

**Affiliations:** 1 Center for Population Health and Aging, Texas A&M University, College Station, Texas; 2 Department of Environmental and Occupational Health, School of Public Health, Texas A&M University, College Station, Texas; 3 Department of Health Promotion and Behavior, College of Public Health, The University of Georgia, Athens, Georgia; 4 Institute of Gerontology, College of Public Health, The University of Georgia, Athens, Georgia; 5 School of Social Work, The University of Georgia, Athens, Georgia; 6 Office of Communications and Creative Services, College of Agricultural and Environmental Sciences, The University of Georgia, Athens, Georgia; 7 Department of Foods and Nutrition, College of Family and Consumer Sciences, The University of Georgia, Athens, Georgia; 8 Department of Kinesiology, College of Education, The University of Georgia, Athens, Georgia; 9 Department of Psychology, Georgia Institute of Technology, Atlanta, Georgia; 10 College of Family and Consumer Sciences, The University of Georgia, Athens, Georgia

## Abstract

**Introduction:**

Given the rising prevalence of obesity in the United States, innovative methods are needed to increase physical activity (PA) in community settings. Evidence suggests that individuals are more likely to engage in PA if they are given a choice of activities and have support from others (for encouragement, motivation, and accountability). The objective of this study was to describe the use of the online Walk Georgia PA tracking platform according to whether the user was an individual user or group user.

**Methods:**

Walk Georgia is a free, interactive online tracking platform that enables users to log PA by duration, activity, and perceived difficulty, and then converts these data into points based on metabolic equivalents. Users join individually or in groups and are encouraged to set weekly PA goals. Data were examined for 6,639 users (65.8% were group users) over 28 months. We used independent sample *t* tests and Mann–Whitney *U* tests to compare means between individual and group users. Two linear regression models were fitted to identify factors associated with activity logging.

**Results:**

Users logged 218,766 activities (15,119,249 minutes of PA spanning 592,714 miles [41,858,446 points]). On average, group users had created accounts more recently than individual users (*P* < .001); however, group users logged more activities (*P* < .001). On average, group users logged more minutes of PA (*P* < .001) and earned more points (*P* < .001). Being in a group was associated with a larger proportion of weeks in which 150 minutes or more of weekly PA was logged (B = 20.47, *P* < .001).

**Conclusion:**

Use of Walk Georgia was significantly higher among group users than among individual users. To expand use and dissemination of online tracking of PA, programs should target naturally occurring groups (eg, workplaces, schools, faith-based groups).

## Introduction

Evidence supports the importance of physical activity (PA) in the prevention of chronic diseases (1–4). Despite the known benefits of PA, only about half of adults meet the Surgeon General’s guideline of 150 minutes of moderate PA weekly (4). Many public health interventions exist to increase PA; however, engagement depends on various factors (5). Personal motivation and activity choice play a major role in behavior change, and methods to increase motivation is a large field of study (6).

Individuals are more likely to be active when they are able to select the types of activities they perform (7). This ability to choose makes the activity more pleasurable and increases the likelihood that a routine will be established (7). In interventions to increase PA, providing more choices promotes the inclusion of people with less common preferences (8).

Website-delivered PA interventions can improve PA among users relative to nonusers (9), and tracking of PA is associated with increased PA (10). Tracking activities may encourage individuals to exercise by helping them to visualize their progress. Numerous consumer technologies allow individuals to track their PA, and these technologies are increasing general awareness about PA engagement (11). The proliferation of exercise technology, such as wearables and other PA tracking smartphone applications, has created a pool of users interested in and conscious about their PA.

Although PA can be an individual activity, group-based PA incorporates a social element to promote engagement (12). Interaction with others can increase engagement in PA because it keeps people encouraged, motivated, and accountable (5,13). The objectives of this study were to 1) examine use of the Walk Georgia platform according to whether the user was registered as an individual or group user and 2) identify factors associated with increased use of the platform among users.

## Methods

### Walk Georgia

Walk Georgia is The University of Georgia (UGA) Cooperative Extension’s free, online PA program designed to encourage PA through community and accountability (14). Users can create an account and track their PA from a computer or mobile phone. The system enables users to set PA goals, track progress, and create custom group challenges. Research-based information about nutrition, PA, health, and behavior change are provided through the program’s blog, a weekly email newsletter, social media outreach efforts, and website.

Walk Georgia was developed in 2008. The first iteration was a PA monitoring program designed as an 8-week campaign occurring once annually (15). It was designed collaboratively by UGA staff, county-based faculty, registered dietitians, health experts, and representatives from other Extension-based walking programs (ie, in Texas and Tennessee). As Extension agents reported the growing popularity of the program, the frequency of offering the 8-week program increased to twice annually and the duration was expanded to 12 weeks. By 2015, the program was expanded to facilitate year-round PA. 

The newest and most advanced online platform was launched in February 2015. To develop the new platform, a program coordinator, web developer, web designer, and public relations specialist (who served as a content manager, editor, and help desk manager) were hired. The 1-year cost of developing the system was approximately $300,000 and included staff salaries, web server space and infrastructure support, software, and online project management tools. The estimated ongoing annual cost for the platform is approximately $30,000, which includes a percentage of staff time, software licenses, web server maintenance, and online subscription tools.

Key features of the system include a simple, mobile-friendly interface (Figure) that allows users to customize their log-in page (eg, upload a profile picture), a goal-setting platform, and a virtual map of Georgia. An incentive for using the platform, the map allows users to “unlock” counties after earning a certain number of points to learn about county facts and resources (eg, county seat, population, top commodities, farmers markets, annual events, nearby state parks or historic sites). The platform also enables users to create PA goals based on recommended levels of weekly PA and subscribe to a blog and weekly email newsletter to receive fitness tips, view healthy recipes, learn about fitness-related events, and post responses on social media (eg, Facebook, Twitter). Participants can log individual PA as well as organize and join groups based on their location (eg, county, city), affiliation (eg, workplace, school, faith-based group), or social group (eg, friends, clubs).

**Figure:**
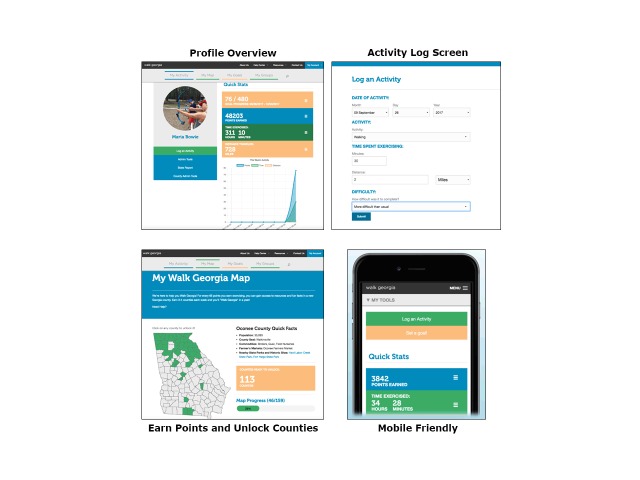
Walk Georgia platform and features.

The Walk Georgia platform is a tracking system and not a formal intervention. Potential users were recruited through sponsored events across the state. Events varied in their target audiences. Some events (eg, state or county fair) were tailored toward individual users, and others were tailored toward group users (eg, state departments of public health to bolster existing workplace wellness activities). The mode of delivery was the same for all users. Users logged into the system and tracked their activity. They did not attend a program physically or virtually. Although users could join groups, the system does not collect information on whether or not group users ever actually meet as a group. Because Walk Georgia is an internet-based tracking system, group members did not have to be in the same geographic area. Group members could exercise independently and still log their activity as part of a group.

### Measures

To examine the use of the Walk Georgia tracking system, we extracted data on user type, group type, user profiles, and PA tracking from the newest platform from February 1, 2015, through June 21, 2017. The institutional review board at The University of Georgia approved all study procedures.

**User type.** Each Walk Georgia participant registers in the system as an individual user. Then they can decide to join one or more groups that include other users. For this study, we categorized users as individual users (ie, those who never joined a group) and group users (ie, those who joined one or more groups).

**Group type.** We classified each Walk Georgia group as one of 7 types: county, city, community, faith-based group, school, workplace, or other. County groups were primarily affiliated with the local county government as part of workplace wellness activities. These groups were typically supported by local Extension offices and included municipal campaigns that targeted county government department personnel. City groups were associated with local city and elected officials as part of workplace wellness activities. Community groups included organizations such as local YMCAs or senior centers. Faith-based groups were religious organizations. Schools groups included elementary, middle, and high schools (public and private) that encouraged PA among students, teachers, staff, and parents. Workplace groups were worksites that promoted PA among their employees, volunteers, customers, advocates, and stakeholders. Other groups were those that did not fall into any of the aforementioned categories (eg, families, weight-loss groups, mall-walking groups).

**User profile.** We documented the number of days since a user created an account (range, 1–970 d) and the number of activities a user logged in the system since creating an account (range, 1–2,602 activities).

**PA tracking**. When users log their PA in the system, they are asked to report the following: type of activity (ie, 70 distance- and non-distance–based activities such as walking, swimming, tennis, or gardening), the duration of activity (tracked in minutes), the distance traveled during the activity (in miles, if appropriate), and the perceived difficulty of completing that activity for the associated duration (on a 5-point Likert-type scale from 1 [easy] to 5 [difficult]). Because not all activities in the Walk Georgia system are distance-based, the system generated points according to metabolic equivalents of task (METs) (16). MET estimates, based on the *2011* *Compendium of Physical Activities,* were assigned to each activity and then incorporated into an equation to award participants points each time they logged activities ([time in minutes × MET estimate] + perceived difficulty) (16).

We tracked average weekly PA logging activity. More specifically, we tabulated the average number of weekly PA minutes logged in the system and the number of weeks a user logged 150 minutes or more of PA. The proportion of weeks a user logged 150 minutes or more of PA was calculated twice: once based on the number of weeks the user had been in the system and once based on the number of weeks the user had logged PA in the system.

**Logged activities**. We documented and ranked the top 20 activities among individual and group users.

### Statistical analyses

We analyzed data by using SPSS version 24 (IBM Corp). We tabulated descriptive statistics for all variables of interest. Both parametric (ie, independent sample *t* tests) and nonparametric (ie, Mann–Whitney *U* tests) tests were used to compare characteristics of website activity between individual users and group users. Two linear regression models were fitted. One model identified factors associated with more logging activity (ie, total number of activities logged) and the other model identified factors associated with a larger proportion of weeks that users logged 150 minutes or more of PA relative to the number of weeks that users had logged PA in the system.

## Results

From February 1, 2015 through June 21, 2017, 13,902 accounts were created in Walk Georgia. Of these 13,902 accounts, 58.6% were individual users and 41.4% were group users. Of the 6,639 (47.8%) accounts with at least one logged activity, 218,766 activities were logged, accounting for 15,119,249 minutes of PA, 592,714 miles, and 41,858,446 points. Of these 6,639 users, 2,271 (34.2%) were individual users and 4,368 (65.8%) were group users. Approximately 95% (4,133 of 4,368) of group users were affiliated with a group when they registered. Among the 233 group users who initially registered as individual users, the average time for them to join a group was 200.4 days. Overall, 21.9% of users resided in rural counties, and a significantly larger proportion of individual users (27.7%) than group users (19.0%) resided in rural areas.

Among the 6,639 accounts with logged activity, on average, group users had created accounts more recently (591 [median, 513] days) than individual users (700 [median, 750] days) (Table 1). However, group users logged significantly more activities (40 [median, 18] activities) than individual users (20 [median, 4] activities). On average, compared with individual users, group users engaged in significantly more minutes of PA, earned significantly more points, rated higher difficulty levels of PA, and logged more weeks with 150 minutes or more of PA. Additionally, the percentage of weeks with 150 minutes or more of logged PA (for weeks with logged activity) was higher among group users (53%) than among individual users (32%).

**Table 1:** Characteristics of Users Who Had Logged ≥1 Activity in Walk Georgia, an Online Physical Activity Tracking Platform, and Differences Between Individual Users and Group Users, February 1, 2015, to June 21, 2017^a^

Characteristic	Total, Mean (SD) [Median] (n = 6,639)	Individual, Mean (SD) [Median] (n = 2,271)	Group, Mean (SD) [Median] (n = 4,368)	*P* Value for* t* Test	*P* Value for Mann–Whitney *U* Test
No. of days since account was created	628 (253) [611]	700 (253) [750]	591 (245) [513]	<.001	<.001
No. of weeks since account was created	89 (36) [87]	100 (36) [107]	84 (35) [73]	<.001	<.001
No. of weeks with activities logged	5.6 (6.3) [3]	3.8 (6.0) [2]	6.5 (6.2) [5]	<.001	<.001
Total no. of activities logged	33 (71) [11]	20 (80) [4]	40 (65) [18]	<.001	<.001
Total no. of minutes engaged in PA	2,277 (13,115) [550]	1,236 (5,743) [170]	2,819 (15,603) [902]	<.001	<.001
Total no. of miles traveled^b^	89 (1,325) [12]	46 (311) [4]	112 (1,617) [21]	.01	<.001
Total no. of points earned	6,305 (57,685) [1,306]	3,085 (14,587) [393]	7,979 (70,279) [2,212]	<.001	<.001
Average no. of minutes engaged in PA	71 (559) [45]	80 (932) [42]	67 (153) [48]	<.001	<.001
Average no. of miles traveled^b^	5.3 (153) [1]	5.2 (95) [1]	5.3 (175) [1]	.98	<.001
Average no. of points earned	292 (5,833) [109]	292 (4,242) [94]	292 (6,509) [115]	>.99	<.001
Average difficulty level of logged PA^c^	1.7 (0.7) [2]	1.6 (0.7) [1]	1.7 (0.7) [2]	<.001	<.001
Average no. of weekly minutes of PA logged	70 (568) [41]	81 (937) [40]	64 (186) [42]	.40	<.001
No. of weeks with ≥150 minutes of PA logged	3.5 (5.4) [1]	1.9 (4.9) [0]	4.3 (5.5) [2]	<.001	<.001
Percentage of weeks with ≥150 minutes of PA logged (for weeks in system)	4.6 (7.0) [2]	2.2 (4.9) [0]	5.9 (7.5) [3]	<.001	<.001
Percentage of weeks with ≥150 minutes of PA logged (for weeks with logged activity)	46 (40) [50]	328 (40) [0]	53 (38) [57]	<.001	<.001

Abbreviation: PA, physical activity.

a During study period, 13,902 accounts were created; of these, 6,639 had logged ≥1 activity. Each Walk Georgia participant registers in the system as an individual user. Then they can decide to join one or more groups that include other users. We categorized users as individual users (ie, those who never joined a group) and group users (ie, those who joined one or more groups).

b Not relevant for all PA activities.

c 5-point Likert-type scale from 1 (easy) to 5 (difficult).

The leading 20 activities for each set of users comprised 24 unique activities (Table 2). Overall, 218,766 activities were logged, of which 79.5% were logged by group users. The 6 most frequently logged activities for both groups were walking, running or jogging, cleaning, weight lifting, active stretching, and cardio sessions. Walking was the leading activity for both sets of users. Four activities were unique to individual users (ballet, CrossFit, sweeping, and calisthenics), and 4 activities were unique to group users (stair climbing, child care, squats, and Zumba).

**Table 2:** Leading Activities Logged by Individual and Group Users of Walk Georgia, an Online Physical Activity Tracking Platform, February 1, 2015, to June 21, 2017^a^

Activity	Group (n = 173,873 Logs)		Individual (n = 49,139 Logs)	
	Rank	No. (%) of Users	Rank	No. (%) of Users
Walking	1	76,217 (43.8)	1	22,270 (45.3)
Running or jogging	2	12,104 (7.0)	2	2,901 (5.9)
Cleaning	3	9,544 (5.5)	5	1,794 (3.7)
Weight lifting	4	8,978 (5.2)	4	2,216 (4.5)
Cardio session	5	4,880 (2.8)	6	1,715 (3.5)
Active stretching	6	4,383 (2.5)	3	2,735 (5.6)
Yard work	7	3,928 (2.3)	9	1,082 (2.2)
Biking	8	3,847 (2.2)	7	1,183 (2.4)
Stair climbing	9	3,818 (2.2)	—	—
Elliptical	10	3,743 (2.2)	11	838 (1.7)
Circuit training	11	2,722 (1.6)	13	748 (1.5)
Swimming	12	2,647 (1.5)	18	502 (1.0)
Child care	13	2588 (1.5)	–	—
Yoga	14	2,387 (1.4)	12	819 (1.7)
Dancing (aerobic)	15	2,319 (1.3)	17	532 (1.1)
Sit-ups	16	2,196 (1.3)	16	571 (1.2)
Gardening	17	1,918 (1.10	14	599 (1.2)
Vacuuming	18	1,876 (1.1)	19	474 (1.0)
Squats	19	1,827 (1.1)	—	—
Zumba	20	1,779 (1.0)	—	—
Ballet	—	—	8	1,117 (2.3)
CrossFit	—	—	10	1,037 (2.1)
Sweeping	—	—	15	584 (1.2)
Calisthenics	—	—	20	407 (0.8)

a Each Walk Georgia participant registers in the system as an individual user. Then they can decide to join one or more groups that include other users. We categorized users as individual users (ie, those who never joined a group) and group users (ie, those who joined one or more groups).

Overall, 571 groups were created with an average of 7.6 members per group (range, 1– 912) (Table 3). The largest proportion (55%) of group users were affiliated with workplace groups (2,405 users; 177 groups; average number of members, 13.6), followed by school groups (791 users; 159 groups; average number of members, 5.0). On average, workplace, county, and city group users logged the most activities in the system, but school group members logged the most minutes. City, faith-based, and workplace group users logged 150 minutes or more of PA for the most number of weeks, on average. However, the proportion of weeks with 150 minutes or more of PA logged was highest among community, city, faith-based, and workplace group users.

**Table 3:** Characteristics of Group Users of Walk Georgia, an Online Physical Activity Tracking Platform, by Type of Group, February 1, 2015, to June 21, 2017^a^

Characteristic	Workplace	County	School	City	Community	Faith-Based	Other	Total
No. of users	2,405	494	791	206	235	75	162	4,368
No. of groups	177	109	159	20	63	11	17	571
Average no. of group members	13.6	4.5	5.0	10.2	3.8	6.8	6.9	7.6
Range of no. of group members	1–912	1–62	1–277	1–47	1–37	1–34	1–94	1–912
No. of days since account was created	537	749	562	640	643	865	783	591
No. of weeks since account was created	76	107	80	91	92	123	112	84
No. of days to join a group since account was created	6.7	17	22	0.3	15	0.2	6.1	10.7
No. of weeks with activities logged	6.9	7.2	5.0	7.5	5.3	7.3	5.2	6.5
Total no. of activities logged	46	42	23	40	31	39	28.9	40
Total no. of minutes engaged in PA	2,871	2,320	3,316	2,702	2,847	2,313	1,481	2,819
Total no. of miles traveled (not relevant for all PA activities)	137	118	62	96	80	66	41	112
Total no. of points earned	8,559	7,631	8,525	6,642	5,837	5,420	3,767	7,979
Average no. of minutes engaged in PA	62	61	80	70	82	58	59	67
Average no. of miles traveled (not relevant for all PA activities)	8.0	2.3	1.9	2.1	2.4	1.6	2.2	5.3
Average no. of points earned	377	170	216	163	182	132	172	292
Average difficulty level of logged PA	1.7	1.7	1.7	1.7	1.6	1.8	1.9	1.7
Average no. of weekly minutes of PA logged	59	57	80	66	78	56	60	64
No. of weeks with ≥150 min of PA logged	4.7	4.7	2.7	5.1	3.8	4.8	2.8	4.3
Percentage of weeks with ≥150 min of PA logged (for weeks in system)	7.3	4.8	3.6	5.7	4.5	4.0	3.4	5.9
Percentage of weeks with ≥150 min of PA logged (for weeks with logged activity)	56	53	41	58	61	58	47	53

a Each Walk Georgia participant registers in the system as an individual user. Then they can decide to join one or more groups that include other users. We categorized users as individual users (ie, those who never joined a group) and group users (ie, those who joined one or more groups).

In the regression model that examined factors associated with more logging activity, users affiliated with a group (B [standard error (SE)] = 21.80 [1.87]; *P* < .001) and users who had accounts for more days (B = 0.02 [0]; *P* < .001), on average, had significantly more PA logging activity. In the regression model that examined factors associated with larger proportions of weeks with 150 minutes or more of logged PA, on average, users affiliated with a group (B = 20.47 [1.03]; *P* < .001) and users who logged activities with higher perceived difficulty (B = 4.45 [0.69], *P* < .001) had significantly larger proportions of weeks with 150 minutes or more of logged PA.

## Discussion

Our results show that group affiliation was associated with more logging activity and more weeks with 150 minutes or more of logged PA. Our findings are consistent with those of other studies that report social motivation contributes to greater participation in and adherence to exercise programs (16–18). Although individual users recorded substantial levels of PA, most Walk Georgia users were group users and logged substantially more activity. Because the Walk Georgia system relies on users to self-report and proactively enter their PA into the system, we do not know whether all PA was accurately logged. Nor do we know whether group users were actually more physically active than individual users or group users were more diligent in their logging activity. Furthermore, users who did not join groups were solely responsible for tracking their activity, whereas group users could designate an individual to log activity for all group members. Regardless, our findings suggest that levels of social accountability (for actual PA and/or system logging) may have been higher among group users than among individual users. Future programs should incorporate the use of wearable PA trackers to more objectively measure the effects of group-based involvement on PA levels and online logging. Additionally, efforts are needed to integrate data collected daily from wearable PA trackers into existing online platforms such as Walk Georgia.

An innovative aspect of Walk Georgia is its assignment of points based on MET estimations (15,16). Users may view points as an incentive for logging activity, and organizations can use points to track and reward users and groups. Individual and group users can visualize and track their weekly and monthly progress and set personal goals to help motivate them to improve performance (18,19). Among group users, an additional incentive exists because each team member can compete to be the leading point earner on their team, and groups can compete with other groups to earn the highest point total. Generally, a point system promotes competition among users and serves as a reward to users (18). The use of a standardized equation that incorporates METs allows all users to engage in the competition equally, not just the walkers, runners, or other users who engage in distance-based activities. This standardized equation also allows those who engage primarily in one type of PA (eg, swimming) to be on a team with those who engage in different activities (eg, tennis, weightlifting, housework). However, MET estimates, supplied by the *2011 Compendium of Physical Activities* (16), were intended to standardize MET intensities for PA but were not intended to be precise PA energy costs per individual (ie, they did not account for “differences in body mass, adiposity, age, sex, efficiency of movement, geographic and environmental conditions in which the activities are performed”) (16).

Given that group users used the Walk Georgia system more than individual users (and had fewer days since accounts were created), we recommend that Walk Georgia and similar programs expand their proportion of group users. This expansion could use strategies to bring together existing members into groups or purposively recruit new users that may inherently join in groups. One strategy could be to identify and recruit naturally occurring groups, organized by location (eg, county, city), affiliation (eg, workplace, school, faith-based), or social group (eg, friends, clubs). Beyond groups such as walking or biking clubs, settings that show promise for purposive Walk Georgia recruitment (as a stand-alone intervention or a component of an existing PA intervention) are faith-based organizations (20), schools (21–23), and worksites (24,25). For example, in schools, Walk Georgia may be particularly attractive because of its interactive map of Georgia, online lesson plans (www.walkgeorgia.org/lesson-plans.php), point calculations, and information on county resources (www.walkgeorgia.org/resources/county-resources.php). These features can be integrated into other curriculum subjects (eg, physical education, math, science, history, social science) (26).

This study had limitations. It was a naturalistic inquiry in which the only data collected were data entered into the Walk Georgia system, which is a tracking system, not a formal intervention. Thus, users were not required to attend sessions, and we do not know whether group users physically or virtually met to engage in PA. Furthermore, data were not collected from users before they registered; thus we could not assess changes over time. Another possible limitation is the limitation of self-reported data: it is important to reiterate that increased activity logging in the system does not necessarily equate to increased engagement in PA (participants could engage in more or less activity than what is being logged in the system). Another limitation was the absence of users’ sociodemographic information (eg, age, sex, race/ethnicity, socioeconomic); such information would allow for comparisons across individual and group users and users in different group types. This limitation prevents us from generalizing our findings beyond this sample. Walk Georgia is beginning to collect these data from users routinely. Structured and comprehensive data collection is recommended for online tracking systems. A small number of groups and organizations, primarily those who received local county Extension sub-awards ($500 awards, which required local matching funds), provided incentives to users. Incentives and recruitment strategies implemented by groups and organizations, not simply group affiliation, could have been the true driver of activity logging. This possible confounder in our study requires further investigation. Future studies should also investigate the activity preferences within group types and identify the influence of the type of PA in which users engage on logging frequency and PA levels (eg, walking vs swimming vs biking). Finally, we do not know whether group users actually competed within or across groups and whether or not this competition influenced their activity logging behavior. Moving forward, Walk Georgia plans to integrate a “leader board” to allow groups and users to view the logged PA of others and formally or informally compete with other users.

Our findings highlight the potential benefit of online PA tracking platforms like Walk Georgia to encourage PA for health promotion and disease prevention. Our analyses suggest such platforms are used more by group users than by individual users; thus, we recommend purposive group user recruitment strategies. The easy-to-use online/mobile-friendly system makes it attractive for diverse audiences despite geographic location. Overall, Walk Georgia provides a way of connecting groups for the common goal of being more physically active. The platform is sufficient to engage users as a proactive activity; however, the system could be enhanced by integrating other passive technologies such as those available via wearables and smartphones.
